# Blame or reflection? Response to Winsberg

**DOI:** 10.1016/j.gloepi.2022.100083

**Published:** 2022-09-09

**Authors:** Peter Streicher, Alex Broadbent

**Affiliations:** a Philosophy Department, Durham University, United Kingdom; b Department of Philosophy, University of Johannesburg, South Africa

We appreciate Eric Winsberg's commentary [1] on our piece ‘Can you lock down in a slum?’ [2]. We take Winsberg to offer a supportive amendment to our paper: that the epidemiological community had a credible claim to expertise in making their recommendations, not shared by non-epidemiologists, which makes epidemiologists especially morally responsible for the negative effects of those recommendations, in contrast to non-experts such as pundits.

This expertise derives, says Winsberg, from epidemiologists' ability to provide models of the consequences of locking down, which constituted evidence against the suggestion that the negative consequences would outweigh the positive, even in contexts where this is highly intuitive: notably, among the very poor. Winsberg provides good examples of model-based arguments directed exactly in this way, as refutations of the intuitive idea that locking down would be untenable and/or ineffective in contexts of global poverty. He identifies a set of arguments that were based on SEIR models specifically designed to show that, even in countries unable to sustain a lockdown, a very large reduction in mortality would be achieved even by locking down for a short period. This is a use of modeling that can fairly be classed as rhetorical, in the non-pejorative sense of being directed primarily or in significant part at persuasion rather than discovery. He identifies another important rhetorical use of modeling too: the argument that a delay long enough for a non-expert to get somewhat to grips with the predictions would cost a very large number of deaths, in respect of which Winsberg coins the nice term “self-recommending”. These arguments, argues Winsberg, were epistemically unwarranted, and this further means that they were morally irresponsible, since they were formulated in ways that prevented an effective evaluation. One might see this as a kind of abuse of epistemic authority. Winsberg concludes that epidemiologists have an even larger proportion of moral responsibility than our paper might imply, because of their special expertise and the use they made of it.

We agree with Winsberg's important points about the use of models. We are more diffident about assigning moral responsibility in quite the way he feels is warranted. In our abstract we say, “This essay contends that (1) some epidemiologists played a central role in formulating and promulgating lockdown as a policy and (2) lockdowns were foreseeably harmful” [2]. Nonetheless, we intended to stop short of making judgements of moral responsibility.

Our goal in the paper was instead to encourage a degree of reflection within the epidemiological community. Epidemiology has a long historical commitment to the plight of the poor. Indeed, many of the paradigm epidemiological episodes (the activities of Ignaaz Semmelweis in the General Hospital of Vienna, the efforts of Austin Bradford Hill in the wool mills around Manchester) have focused on the poor, while many contemporary public health initiatives are strongly focused on the unequal and, it is inferred, unfair impact that many aspects of social organization have on poorer people in wealthy and non-wealthy nations. Thus our objective was not to pass a moral judgment of our own, so much as to prompt an internal reflection among epidemiologists.

It is arguably part of the epidemiological DNA that, once a conclusion is settled on, epidemiological activities should be directed in a rhetorical—that is, a persuasive—way. There is nothing intrinsically wrong with this, given that epidemiology is a science with a social goal. A famous example is a paper in 1959, in which several luminaries assembled a large patchwork of evidence and intellectual skills, in an effort to put to bed the various alternative non-causal hypotheses about the relationship between smoking and lung cancer [3]. This paper was rhetorical, in the sense of being directed at persuasion, rather than reporting the results of a discovery-oriented inquiry. Its structure, its authorship, the nature of its arguments and evidence—all of these things make sense only in the context of a policy debate. But does that mean it was morally problematic? We do not think so. The use of scientific argumentation to drive a policy change is nothing new to epidemiology, and it is hard to imagine epidemiology without it. We do not think (and nor do we say that Winsberg implies) there is anything special about the use of modeling as part of a scientific argument, nor in the fact that it is a kind of argumentation that non-experts may be unable to match.

We suppose Winsberg agrees (or may consistently agree) with our sentiment that scientific argumentation for political ends is sometimes justified. The force of his criticism is that it was not justified in this instance, because the situation was not in fact epistemically (one might say “scientifically”) clear in a way that would warrant it. (That is, there was not in fact adequate evidence, even given the uncertainties of the time, that a lockdown in an impoverished region was the right thing to do, or that the intuitive reasons against it were mistaken.) We agree.

Where we may differ from Winsberg, however, is that we are less confident that moral responsibility can be attributed to epidemiologists for getting this wrong. Stanley Milgram's dramatic experiments on obedience to authority suggest that people will dramatically contravene their own prior moral judgements upon instruction from an authority [4]. We suspect that scientists, who are humans first and foremost, will not readily resist a general sentiment in a community to which they belong. If one is accepts aspects of Thomas Kuhn's views, then it may even be scientific training reinforces this tendency [5].

The reasons for a general sentiment taking the shape within a given community are deeply mysterious. Why did a witch-hunt emerge in Salem in 1692–3? Why do so many ordinary people in so many different times and places commit atrocious acts of genocide in the certain circumstances? These are matters of inquiry for psychologists and sociologists. We cannot say with certainty that, if we had been epidemiologists in 2020, we would have seen things as we in fact did, regarding what we took and take to be the inappropriateness of lockdowns for certain global contexts. Even if we had, we cannot be confident that that we would have spoken up. An epidemiologist who would speak up against the general sentiment at that time was a brave one indeed, and those who did have suffered, even where their scientific credentials were impeccable and where there arguments were at least as cogent as many other arguments circulating at the time. Placing blame for these dynamics, horrible though they are, is difficult when they are so common in human history.

For these reasons we are hesitant to pass a moral judgment, and especially to do so on epidemiologists in general. We agree with Winsberg's insightful critique of the epistemic situation: of his argument, in other words, that certain epidemiologists did very poor science and abused their epistemic authority. We suspect they abused their influence on their own field (a further dimension of development for Winsberg's argument) as well as on non-experts. We also accept that—as in any situation—some of these individuals and groups most probably did err morally, as Winsberg contends. But we do not feel qualified to identify those that erred. And even if we did, we would not extend such a judgment to the epidemiological community as a whole.

What we do believe, however, as in the aftermath of any serious departure from the path directed by one's value system, is that the epidemiological community should reflect on whether what it did is at odds with its purported values. It must do this if it is to make serious efforts to improve future interventions, and if it is serious about its own commitment to addressing health inequalities. The community has thrown its weight behind a global policy that may have done more to grow global health inequalities than any other policy in living memory, for the sake of a benefit that, in our view, it could and should have known was unattainable for the world's poorest people and places. We do not say that the epidemiological community should be held morally responsible for this. Rather, we say that the epidemiological community should reflect deeply on whether there is a tension between the values it purports to aspire to and its actions during the Covid-19 pandemic. This reflection should be especially with the global poor in mind, and with an understanding of the way that science can so often be shaped, even unconsciously, by the interests of the wealthy and powerful.

Nonetheless, even this more modest message is one we fear is not being heeded. None of us likes to admit error, or even reflect on its possibility. Winsberg's argument and ours come together again in holding that, in this instance, it would be morally blameworthy not to do so.

## Author note

No grant supported this research.
